# Spectrum Situation Awareness for Space–Air–Ground Integrated Networks Based on Tensor Computing

**DOI:** 10.3390/s24020334

**Published:** 2024-01-05

**Authors:** Bin Qi, Wensheng Zhang, Lei Zhang

**Affiliations:** 1 Shandong Provincial Key Laboratory of Wireless Communication Technologies, School of Information Science and Engineering, Shandong University, Qingdao 266237, China; qib@mail.sdu.edu.cn; 2 Shanghai Research Institute of Intelligent Autonomous Systems, Tongji University, Shanghai 200092, China; reizhg@tongji.edu.cn

**Keywords:** space–air–ground integrated networks, spectrum situation awareness, tensor computing, tensor eigenvalue

## Abstract

The spectrum situation awareness problem in space–air–ground integrated networks (SAGINs) is studied from a tensor-computing perspective. Tensor and tensor computing, including tensor decomposition, tensor completion and tensor eigenvalues, can satisfy the application requirements of SAGINs. Tensors can effectively handle multidimensional heterogeneous big data generated by SAGINs. Tensor computing is used to process the big data, with tensor decomposition being used for dimensionality reduction to reduce storage space, and tensor completion utilized for numeric supplementation to overcome the missing data problem. Notably, tensor eigenvalues are used to indicate the intrinsic correlations within the big data. A tensor data model is designed for space–air–ground integrated networks from multiple dimensions. Based on the multidimensional tensor data model, a novel tensor-computing-based spectrum situation awareness scheme is proposed. Two tensor eigenvalue calculation algorithms are studied to generate tensor eigenvalues. The distribution characteristics of tensor eigenvalues are used to design spectrum sensing schemes with hypothesis tests. The main advantage of this algorithm based on tensor eigenvalue distributions is that the statistics of spectrum situation awareness can be completely characterized by tensor eigenvalues. The feasibility of spectrum situation awareness based on tensor eigenvalues is evaluated by simulation results. The new application paradigm of tensor eigenvalue provides a novel direction for practical applications of tensor theory.

## 1. Introduction

### 1.1. Space–Air–Ground Integrated Networks and Situation Awareness

The space–air–ground integrated networks (SAGINs), working as network infrastructures, provide ubiquitous, collaborative, and efficient information services for various network applications in large-scale space. The SAGINs are composed of three kinds of networks, including space networks, air networks, and ground networks. The ground networks are the main body of SAGINs. The space networks and air networks serve primarily as supplements and extensions. Through the deep integration of multidimensional networks, the SAGINss can effectively utilize various resources comprehensively, carry out intelligent network control and information processing so as to flexibly cope with the network services with different demands, and realize the functions of an integrated communication system, which is called communication–computing–cache.

Under the framework of SAGINs, the space networks consist of various satellite systems that form the space-backbone network and space-access network, providing global coverage, ubiquitous connectivity, broadband access, and communication services, mainly for rural areas and the regions where ground networks are difficult to be deployed. The space networks are composed of high-altitude communication platforms and UAV networks, which can enhance coverage, enable edge network access, and provide additional network services in emergency situations. The ground networks are mainly composed of ground internet and mobile communication networks, which are responsible for the network service in the business-intensive areas like cities or areas requiring high communication quality.

#### 1.1.1. Space Networks

The space networks consist of different types of satellites, constellations, and the corresponding ground infrastructures, including the ground stations and the control centers. Satellites can be divided into GEO, MEO, LEO [1], and VLEO [2], according to the altitudes where they are working. GEO satellites, also called high elliptical orbit satellites, work at an altitude of more than 36,000 km and are often used for international long-distance communication. MEO satellites orbit at an altitude of 2000–36,000 km, and LEO satellites orbit at an altitude of 400–2000 km. Because their orbits are relatively low, the former are often used as positioning systems, such as GPS and Beidou navigation satellite systems, while the latter are often used for earth observation, earth surveys, and space stations. VLEO satellites have the lowest orbits, so they can be used to provide high-speed data communication, precise positioning, and other services.

Since satellites have a high position and wide coverage, they are regarded as the main method to enable global mobile communications. Iridium, for example, expects to provide global voice and data communication by adopting VLEO satellites. Starlink creates a low-cost, high-coverage space network that supports global communications and can provide internet access services at the speeds of optical fiber by increasing the density of satellites. Meanwhile, due to the high position of the satellites, transmission links are long and the propagation delays are large, making it easy to be destroyed by random or deliberate nodes attacking of malicious nodes during signal transmission. So, it is difficult to guarantee the QoS of real-time information interactive applications.

#### 1.1.2. Air Networks

The air networks consist of stratospheric airships, high-altitude balloons, drones, helicopters, and other high-altitude platforms. As an intermediate layer for SAGINs, it can provide data routing between the platforms and the ground networks and exchange data information with space networks. High-altitude platforms (HAPs) are the main body of the air networks, which are located 2–20 km above the stratosphere and upper troposphere [3]. HAPs, compared with satellite communication platforms, have the advantages of simple deployment, short communication response time, and low cost and are often used in temporary high-bandwidth communication scenarios, such as emergency communication and disaster relief activities.

However, due to the rapid changes in HAPs network topology and communication links, in order to ensure the security and stability of the network and provide users with high throughput and low delay network access services, it is necessary to design effective coordination mechanisms such as channel allocation, which may result in a complex network structure or allocation mechanism and high allocation cost.

#### 1.1.3. Ground Networks

The ground networks are composed of many sub-networks, such as Wireless Local Area Networks (WLAN), cellular networks, and Ad Hoc Networks. Most of the common communication modes in our daily lives can be classified into the ground networks. Meanwhile, the ground networks can provide users with communication services with high data transmission rates and throughputs.

However, as the construction of base stations and other infrastructure is more limited by complex terrain, the high construction cost leads to poor network services provided by the ground networks in remote areas with sparse populations. In addition, ground infrastructure is vulnerable to extremely severe weather, man-made damage, and other factors, resulting in communication disruption. To sum up, it is difficult to depend on the ground networks singly to satisfy the rapidly increasing and complicating communication needs.

#### 1.1.4. Spectrum Situation Awareness

Spectrum situational awareness originates from spectrum sensing technology and the concept of situational awareness. Spectrum sensing was first proposed in [4], where Mitola coined a new concept of cognitive radio (CR) to realize dynamic spectrum access. In CR networks, the secondary users (SUs) can access the idle channels that are not occupied by the primary users (PUs) to totally improve spectrum efficiency. Spectrum sensing technology can obtain spectrum usage information in wireless systems through various signal detection methods, detect spectrum holes, and prevent interference to the PUs. The concept of situational awareness originated in the 1980s. It is mainly used to analyze information about the environment to acquire current and future situations and make corresponding judgments and decisions. Now, it is extended to perceive the environmental elements in a certain time and a certain space, understand the meaning of these elements and predict their future state.

There is still no exact definition of spectrum situational awareness at the moment. However, according to the core purpose of spectrum sensing and situational awareness, it can be understood as the acquisition of various state information from the current spectrum space, such as spectrum busy state and spectrum radiated power. On this basis, various parameters and the development trend of spectrum space can be analyzed. Its core technologies are summarized as wide-area spectrum situational awareness, dynamic spectrum situation generation, and spectrum situation utilization [5].

### 1.2. Tensor Computing and Tensor Eigenvalues

Tensor computing is a new concept mainly including tensor decomposition, tensor completion, and tensor eigenvalue. Tensor decomposition and completion have been widely used in signal processing [6], machine learning [7], big data analysis [8] and other fields [9,10], while engineering applications of tensor eigenvalue in the field of communications are relatively lacking.

#### 1.2.1. Tensor Decomposition and Tensor Completion

Tensor decomposition originated with Hitchcock in 1927 [11], and the conception of the multiway model was proposed by Cattell in 1944 [12]. It can be viewed as the higher-order generalization of matrix factorization, that is, converting higher-order data into a combination of lower-order and lower-dimension data. The two most common types of tensor decomposition are CP decomposition and Tucker decomposition.

Based on the definition of the rank-one tensor, Hitchcock proposed that the tensor was divided into a finite number of rank-one tensors for the first time, named as polyadic form, which was the rudiments of CP decomposition. The name of CP decomposition has not been clearly given; parallel factors [13] and CANDECOMP [14] both were the former names of CP decomposition until Kiers called this form of tensor representation method CANDECOMP/PARAFAC: that is, CP decomposition [15]. For example, given a third-order tensor $X\in R^{I\times J\times K}$, it can be re-written by CP decomposition as

(1) $$X=\sum_{r=1}^{R}a_{r}\circ b_{r}\circ c_{r},\;$$

where the positive integer *R* is called CP-rank, and $a_{r}\in R^{I},b_{r}\in R^{J}$, and $c_{r}\in R^{K}$ for r=1,…,R; the notation “∘” represents the outer product.

Tucker decomposition, first proposed by Tucker in 1963 [16], can be regarded as a higher-order extension of principal component analysis (PCA). Like CP decomposition, Tucker decomposition also has many other names, such as three-mode PCA (3MPCA) [17] and higher-order SVD (HOSVD) [18]. For example, for a third-order tensor $X\in R^{I\times J\times K}$, we can rewrite it by Tucker decomposition as

(2) $$\begin{array}{cc} X & =G\times_{1}A\times_{2}B\times_{3}C=\sum_{l=1}^{L}\sum_{m=1}^{M}\sum_{n=1}^{N}G_{lmn}a_{l}\circ b_{m}\circ c_{n}=\sum_{l=1}^{L}\sum_{m=1}^{M}\sum_{n=1}^{M}G_{lmn}A_{il}B_{jm}C_{kn}, \end{array}\;$$

where $A\in R^{I\times L},B\in R^{J\times M}$, and $C\in R^{K\times N}$ are factor matrices. The tensor $G\in R^{L\times M\times N}$ is called the core tensor of Tucker decomposition, and $a_{l}\in R^{I},b_{m}\in R^{J}$ and $c_{n}\in R^{K}$ for l=1,…,L,m=1,…,M,n=1,…,N. The notation “$\times_{n}$” represents the n-mode product.

Tensor completion is used to complete the tensor by estimating the values according to the relationship between the existing data, structural properties of data, and the missing elements, which is often used in pattern recognition [19], compressed sensing [20] and other fields. Tensor completion is mainly divided into two kinds of methods [21]. One is based on the low-rank property, called the nuclear norm minimization method, like [22,23]. The other is based on low-rank tensor decomposition, like [24,25].

#### 1.2.2. Tensor Eigenvalue

Tensor eigenvalue is the essential part of tensor computing, which is developed from the concept of matrix eigenvalue. Unlike a singular value or eigenvalue after the matricization of a tensor, which is a matrix singular value or eigenvalue, respectively, here, we discuss the tensor eigenvalue, which is calculated by regarding the tensor as a whole unit. For different research purposes and application backgrounds, scholars have put forward many different concepts of tensor eigenvalue, but the origin of tensor eigenvalue was proposed by professors Qi and Lim based on their respective research directions independently. The former treats the tensor eigenvalues as roots of multidimensional polynomials [26], while the latter proposed the concept of tensor eigenvalue by analogy with the Rayleigh quotient of symmetric matrix eigenvalues and the constraint variational method [27]. Although their starting points are different, the two concepts are essentially the same. Since the introduction of tensor eigenvalues, with a particular focus on Z-eigenvalues, they have been extensively utilized in a wide range of fields. These applications include global optimization problems [28,29], hypergraph theory [30,31], homogeneous polynomial system stability problems [32], and many others.

For supersymmetric tensors, where tensor elements have the same value if their indexes belong to the full permutation set of indexes, Qi called a number λ an N-eigenvalue of $A\in R^{\left[m,n\right]}$ if λ is the solution of the following homogeneous polynomial equation

(3) $$Ax^{m-1}=\lambda x^{\left[m-1\right]},$$

where ${\left(Ax^{m-1}\right)}_{i}=\sum_{i_{2},\ldots ,i_{m}=1}^{n}A_{i,i_{2},\ldots ,i_{m}}x_{i_{2}}\cdots x_{i_{m}}$ and $x^{\left[m-1\right]}={\left[x_{1}^{m-1},x_{2}^{m-1},\cdots ,x_{n}^{m-1}\right]}^{T}$, and he called the solution x an N-eigenvector of A associated with the N-eigenvalue λ. If $\lambda \in R,x\in R^{n}$, λ is called an H-eigenvalue and x is called an H-eigenvector. Lim called them $l^{m}$-eigenvalue and $l^{m}$-eigenvector, which were defined as follows

(4) $$A\left(I_{n},x,\ldots ,x\right)=\lambda x^{m-1},\;$$

where $I_{n}\in R^{n}$ is an all-one vector. E-eigenvalues and Z-eigenvalues satisfy

(5) $$\left\{\begin{array}{c} Ax^{m-1}=\lambda x\\ x^{T}x=1 \end{array}\right.,\;$$

where if $\lambda \in R,x\in R^{n}$, λ is called the Z-eigenvalue; otherwise, λ is called the E-eigenvalue.

In order to solve the problem of eigenvalue computing of non-symmetric tensors and different structure tensors as well as summarize different types of tensor eigenvalues, B-eigenpairs and $B_{R}$-eigenpairs [33,34], where they are called $B_{R}$-eigenpairs if B-eigenpairs are all real, are proposed and defined as

(6) $$\left\{\begin{array}{c} A^{\left\{k\right\}}x^{m-1}=\lambda Bx^{m-1},\;m=m^{\prime };\\ A^{\left\{k\right\}}x^{m-1}=\lambda Bx^{m^{\prime }-1},Bx^{m^{\prime }}=1,\;m\neq m^{\prime }. \end{array}\right.\;$$

Therein, $B\in R^{\left[m^{\prime },n\right]}$ is a symmetric tensor, which has different forms depending on the different types of tensor eigenvalues we want to express. The notation “$A^{\left\{k\right\}}$” means to find the eigenvalues of A in the *k*-th direction. Because A is a non-symmetric tensor, different order directions lead to different eigenpairs, and *k* is needed to indicate the order direction and the B-eigenpair can also be called a mode-k B-eigenpair.

Four types of eigenpairs described above can be represented by B-eigenpair and $B_{R}$-eigenpair. If B is the unit tensor $I\in R^{\left[m,n\right]}$, and $m=m^{\prime }$, the mode-1 B-eigenpairs are the N-eigenpairs and the mode-1 $B_{R}$-eigenpairs are the H-eigenpairs. If B is the unit matrix $I\in R^{n\times n}$, and $m^{\prime }=2$, the mode-1 B-eigenpairs are the E-eigenpairs and the mode-1 $B_{R}$-eigenpairs are the Z-eigenpairs.

The main contributions of the paper can be summarized as follows:

1. A novel tensor-based spectrum situational awareness model is proposed to store and process multidimensional, heterogeneous, and massive spectral big data from space–air–ground integrated networks.
2. Two eigenvalue computing schemes, including the semidefinite relaxation algorithm and the homotopy continuation algorithm, are included to calculate the eigenvalues of spectrum situational awareness tensors. The computing performances of two algorithms are evaluated, and the superiority of the homotopy algorithm for tensor eigenvalue estimation is demonstrated.
3. A novel spectrum situational awareness scheme based on tensor eigenvalues is proposed, where the tensor eigenvalue distribution is used to evaluate the state of the spectrum. The new application paradigm of a tensor eigenvalue provides a novel direction for practical applications of tensor eigenvalues, especially using tensor eigenvalue distributions to construct a spectrum situational awareness scheme.

The remainder of the paper is provided as follows. Section 2 introduces the space–air–ground integrated network model and the tensor-based spectrum situation awareness model. Section 3 introduces the problem of tensor eigenvalue computing and two classical tensor eigenvalue computing algorithms, a semidefinite relaxation algorithm and a homotopy continuation algorithm. The performance of the two algorithms are compared in terms of convergence ratio, accuracy and CPU time. Section 4 introduces a tensor-eigenvalue-based spectrum sensing algorithm and simulation results with theoretical analysis. In Section 5, the paper is concluded.

## 2. System Model

### 2.1. Space–Air–Ground Integrated Network Model

**Figure 1:**
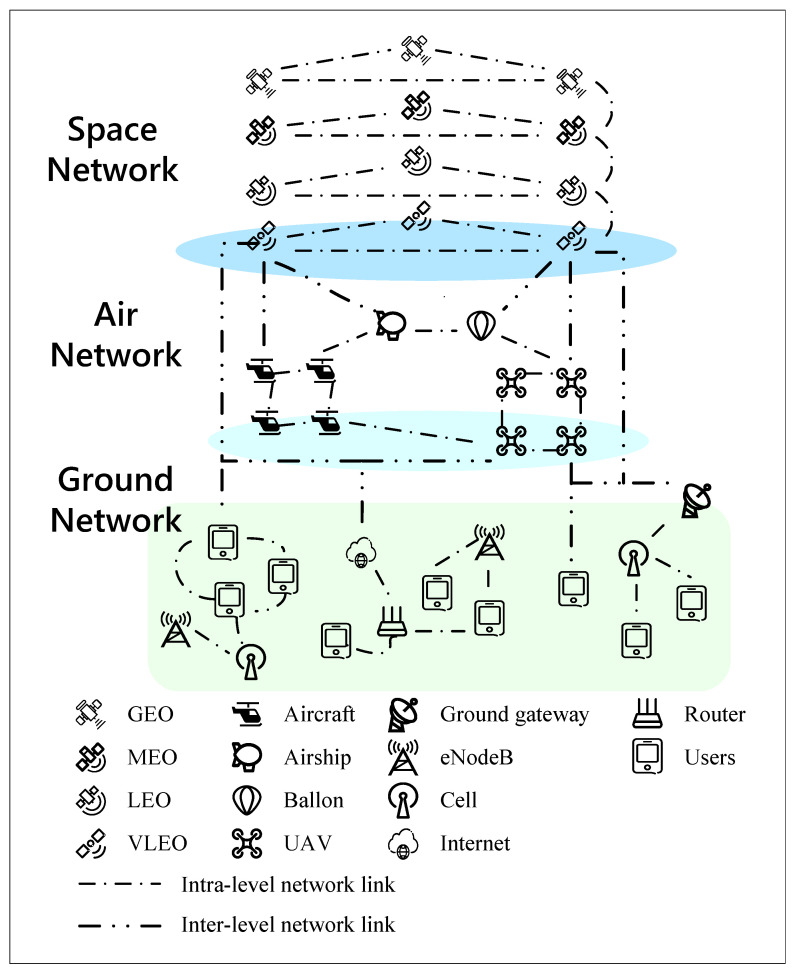
Space–air–ground integrated network model.

The model of SAGINs is shown in Figure 1, where space networks, air networks and ground networks are three layers. As shown in the figure, the satellites including GEO, MEO, LEO and VLEO exchange information and realize key communication functions through intra-segment wireless links. In air networks, high-altitude platforms, such as aircrafts, airships, balloons, and UAVs, constitute corresponding sublayer networks. The air networks can provide special functions for emergency communications and other tasks. In ground networks, 5G networks can provide basic network infrastructures and basic internet access services. Many services of ground networks are implemented through inter-segment communication, like using air networks for Internet access enhancement services in emergency communications and using space networks for global communications through ground gateways.

In this model, sublayers within each network are independent of each other so as to give full play to their characteristics and advantages and facilitate the modification of the topology structure. Inter-segment networks are interwoven and fused with each other to realize the integration of space networks, air networks, and ground networks to complete the core purpose, accurate acquisition, rapid processing, and efficient transmission of information. A whole model and unified heterogeneous big data model are required!

### 2.2. Tensor-Based Spectrum Situation Awareness Model

For the proposed unified SAGINs model, the corresponding big data model is required to store and process heterogeneous big data. We try to propose a spectrum situational awareness model, adopt the unified data tensor model to solve the above problem, and use the data of this model to deal with the spectrum utilization issues from the perspective of spectrum usage.

For multidimensional, heterogeneous, and massive spectral big data, each order of tensors is used to represent a specific dimension, such as time, space, and frequency. The smallest unit of the tensor is constant; that is, the element of the tensor whose index is greater than or equal to the order is constant. In practical application, it is often necessary to collect multiple data in one location. Since these data do not belong to the same variable, they are often placed separately in multiple tensors of the same size during tensor modeling; that is, tensors are added to these data, respectively. Different tensors are represented by different notations, resulting in heterogeneous data. The huge amount of symbols and the heterogeneous data tensors pose a challenge to the final form of the same representation of big data.

To overcome this challenge, the tensor representation model of vector elements is proposed. Using vectors instead of scalars as the smallest unit of the tensor, all information can be expressed without error as long as the vector elements are ordered in the same order. The tensor representation model of vector elements is represented by the symbol $A_{i_{1}i_{2}\cdots i_{m}\left(j\right)}$, where m is the order of tensor A. For example, the temperature and humidity in a certain space are measured, and three sampling points are taken for each length, width, and height. The tensor model is a three-order three-dimensional tensor A with two variables, and the symbol is denoted as $A_{xyz(a)}$, where $A_{xyz}=\left(t,h\right)$ and x, y, z, t, and h represent the length, width, height, temperature, and humidity, respectively. *a* is the sequence number of the variable in the vector. $A_{xyz(1)}$ represents the temperature tensor of the space and $A_{111(1)}$ represents the specific temperature value at sampling point 111. In other words, the uniform variable is formed as the order of the tensor, and the remaining variables are converted into vectors and stored in the tensor.

**Figure 2:**
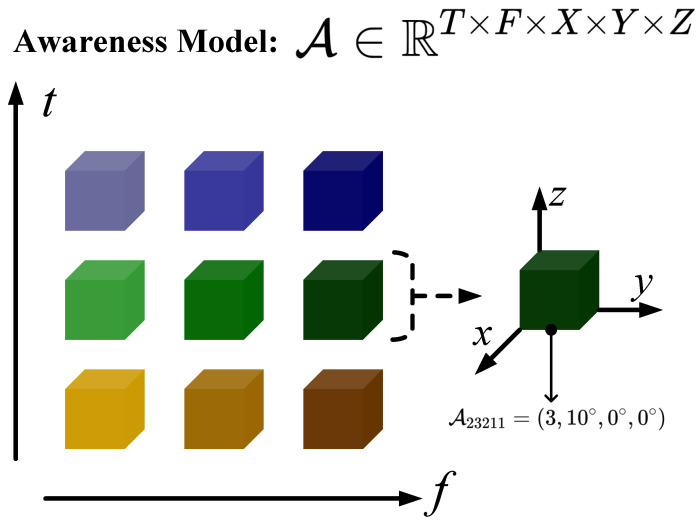
Tensor representation model and tensor-based spectrum situation awareness model.

Using the tensor representation model of vector elements above, we can obtain the tensor-based spectrum situation awareness model. For the space–air–ground integrated spectrum situational awareness, user-centered situational awareness is carried out, and the awareness model is a five-order tensor composed of time, frequency, longitude, latitude, and altitude, where the elements of the tensor are data vectors. A five-order tensor $A\in R^{T\times F\times X\times Y\times Z}$, composed of time, frequency, longitude, latitude, and altitude, is taken as an example of the awareness model here, and the model is shown in Figure 2. The data in the tensor A represent the variation of the data along longitude, latitude, and altitude at different frequencies measured at different times. Taking $A_{23211}$ for example, where $A_{23211}=\left(3,10{}^{\circ},0{}^{\circ},0{}^{\circ}\right)$, it means that at the first sampling time, the sampling frequency, sampling longitude, sampling latitude, sampling altitude, and radiation signal intensity are 3, the yaw angle is 10°, and the pitch and roll angles are 0°.

## 3. Tensor Eigenvalue Calculation

### 3.1. Related Work of Tensor Eigenvalue Calculation

Unlike matrix eigenvalue generation, the eigenvalue calculation problem of third or higher-order tensors can be considered as an NP-hard problem [35] due to the so-called “curse of dimensionality”. Nonetheless, several algorithms computing one or some eigenvalues of a tensor have been developed recently. Most algorithms are flawed, and these algorithms are designed for tensors of specific types, such as non-negative or real symmetric tensors.

For non-negative tensors, Ng, Qi, and Zhou proposed a power-type method to compute the largest H-eigenvalue of a non-negative tensor, which was called the Ni-Qi-Zhou method based on the Perron–Frobenius theorem [36]. For real symmetric tensors, Hu, Huang, and Qi proposed a sequential semidefinite programming method for computing extreme Z-eigenvalues [37]. Hao proposed a sequential subspace projection method for a similar purpose [38]. Kolda and Mayo proposed a shifted power method (SSHOPM) for computing Z-eigenvalues [39]. Han proposed an unconstrained optimization method for computing a real generalized eigenvalue for every order real symmetric tensor [40]. Lv and Ma proposed a Levenberg–Marquardt method to obtain all the H-eigenvalues of real semi-symmetric tensors [41]. In addition, for all symmetric tensor eigenvalues, there are many tensor eigenvalue algorithms such as NCM (Newton correction method), O-NCM [42] and FNS (fast Newton–Shultz-type iterative method) [43].

In recent years, the research on the eigenvalue computing of general tensors has made a breakthrough. For general tensors, Cui, Dai, and Nie proposed a novel method for computing all real eigenvalues of symmetric tensors by semidefinite relaxation [33] and then extended it to general tensors [44]. Chen, Han, and Zhou also proposed a homotopy method for computing all eigenvalues [34]. For solving tensor equations with applications, Liang, Zheng, and Zhao proposed alternating iterative methods based on ADMM, such as G-ADMM (Gauss–Seidel scheme) and TT-ADMM (tensor–train) [45]. Chen et al. provided a new idea to compute tensor eigenvalues by using digital signal processing technology and proved its feasibility from the perspective of a continuous-mode system [46].

To sum up, since the concept of the tensor eigenvalue was proposed, scholars have paid much attention to solving the tensor eigenvalue problem and put forward a mass of tensor eigenvalue numerical approximate algorithms, but most of them are based on the Newton iteration method to estimate values, just changing the initial conditions or the iteration equation to accelerate the rate of equation convergence, and a few use convex optimization algorithms to solve eigenvalue problems with special structures.

### 3.2. Tensor Eigenvalue Calculation Algorithms

#### 3.2.1. Semidefinite Relaxation Algorithm

The semidefinite relaxation method, which is one of the first few algorithms to try to compute all eigenvalues of a tensor, is of great significance for the proposal and improvement of the subsequent algorithm, although it has some deficiencies like low convergence rate, long convergence time, inability to compute complex eigenvalues, and others. For the semidefinite relaxation method, to calculate Z-eigenvalues or E-eigenvalues, the corresponding algorithm is different, but the main idea is identical. Below, we take its Z-eigenvalue algorithm as an example to introduce the semidefinite relaxation method.

Let a eigenpair (λ,x) be a Z-eigenpair of $A\in R^{\left[m,n\right]}$. We can derive that Z-eigenvalue λ is $Ax^{m}$ and the corresponding eigenvector x needs to satisfy $Ax^{m-1}=\left(Ax^{m}\right)x,x^{T}x=1$. So, we can obtain a polynomial function $p_{z}$

(7) $$p_{z}\left(x\right)=\left(Ax^{m-1}-\left(Ax^{m}\right)x,x^{T}x-1\right).$$

Then, x is a Z-eigenvector of A when $p_{z}\left(x\right)=0$. The Z-eigenvalues can be calculated from the smallest to the largest.

Firstly, compute the smallest Z-eigenvalue $\lambda_{1}$ of A. Define the polynomial optimization problem

(8) $$\min f\left(x\right):=Ax^{m}\;\;s.t.\;p_{z}\left(x\right)=0,$$

where the optimal solution of (8) is $\lambda_{1}$. According to Lasserre’s hierarchy [47], using the semidefinite relaxation method, the polynomial optimization problem (8) can be converted to

(9) $$\left\{\begin{array}{c} f_{1}^{k}=\min\langle f,y\rangle \\ \;s.t.\;\langle 1,y\rangle =1,L_{p}^{\left(k\right)}\left(y\right)=0,M_{k}\left(y\right)⪰0, \end{array}\right.$$

where 〈·〉, y, $L_{p}^{\left(k\right)}\left(y\right)$, and $M_{k}\left(y\right)$ are defined in [47]. Then, with $k=k_{0}$ as the initial point, the solutions of the semidefinite relaxation (9) can be obtained, where $k_{0}=\left\lceil \left(m+1\right)/2\right\rceil$. If there is no solution for $k=k_{0}$, A has no Z-eigenvalue; otherwise, solve (9) again with an optimizer $y^{*}$. If $y^{*}$ satisfies $\mathrm{rank}M_{t-k_{0}}\left(y^{*}\right)=\mathrm{rank}M_{t}\left(y^{*}\right)$, we obtain $\lambda_{1}=f_{1}^{k}$; otherwise, let k=k+1 and repeat the above procedures. In the end, we can obtain the smallest Z-eigenvalue $\lambda_{1}$.

Secondly, we need to know whether the next larger Z-eigenvalue $\lambda_{i+1}$ exists or not and then compute $\lambda_{i+1}$ if it exists. Let δ be a positive number that is close to zero. To find larger eigenvalues, (9) is modified to the following formula

(10) $$\min f\left(x\right)\;s.t.\;p_{z}\left(x\right)=0,\;f\left(x\right)\geq \lambda_{i}+\delta .\;$$

Like (9), we can obtain Lasserre‘s hierarchy of semidefinite relaxations

(11) $$\left\{\begin{array}{c} f_{i+1}^{k}=\min\langle f,y\rangle \\ \;s.t.\;\langle 1,y\rangle =1,\;L_{p}^{\left(k\right)}\left(y\right)=0,\;M_{k}\left(y\right)\geq 0,\;L_{f-\lambda_{i}-\delta }^{\left(k\right)}\left(y\right)⪰0. \end{array}\right.$$

If the semidefinite relaxation (11) converges, which is checked by condition (10), the Z-eigenvectors $x_{1},\ldots ,x_{r}$ can be computed by the method in [48]. Then, consider the optimization problems

(12) $$\left\{\begin{array}{c} v^{+}\left(\lambda_{i},\delta \right):=\max f\left(x\right)\\ s.t.\;p_{z}\left(x\right)=0,\;f\left(x\right)\leq \lambda_{i}+\delta . \end{array}\right.$$

and

(13) $$\left\{\begin{array}{c} v^{-}\left(\lambda_{i},\delta \right):=\min f\left(x\right)\\ s.t.\;p_{z}\left(x\right)=0,\;f\left(x\right)\geq \lambda_{i}-\delta . \end{array}\right.$$

The solutions of (12) and (13) are used to determine whether the eigenvalue is an isolated eigenvalue in order to generate δ, which is used in (11). $\lambda_{i}$ is an isolated Z-eigenvalue of A in $\left[\lambda_{i}-\delta ,\lambda_{i}+\delta \right]$ if and only if

(14) $$v^{-}\left(\lambda_{i},\delta \right)=v^{+}\left(\lambda_{i},\delta \right).\;$$

The implementation of the algorithm is shown in Algorithm 1 [44].
**Algorithm 1** Compute all Z-eigenvalues of $A\in R^{\left[m,n\right]}$Step 0. Let $k=k_{0}$, with $k_{0}:=\left\lceil \left(m+1\right)/2\right\rceil$.Step 1. Solve the semidefinite relaxation (9) by using Sedumi [49]. If there is no solution, A has no Z-eigenvalue and stop; if not, compute a minimizer $y^{*}$.Step 2. If $\mathrm{rank}M_{t-k_{0}}\left(y^{*}\right)\neq \mathrm{rank}M_{t}\left(y^{*}\right)$ for every t≤k, let k=k+1 and return to Step 1; if not $\lambda_{1}=f_{1}^{k}$, set i=1.Step 3. Let δ=0.05.Step 4. Solve (12) and (13) for the optimal solutions $v^{+}\left(\lambda_{i},\delta \right),v^{-}\left(\lambda_{i},\delta \right)$ by using Sedumi. If $v^{+}\left(\lambda_{i},\delta \right)\neq v^{-}\left(\lambda_{i},\delta \right)$, let δ:=δ/5 and compute $v^{+}\left(\lambda_{i},\delta \right)$, $v^{-}\left(\lambda_{i},\delta \right)$ again. Repeat this step until $v^{+}\left(\lambda_{i},\delta \right)=v^{-}\left(\lambda_{i},\delta \right)$.Step 5. Let $k=k_{0}$.Step 6. Solve the relaxation (11) by using Sedumi. If there is no solution, $\lambda_{i}$ is the largest Z-eigenvalue and stop; if not, compute a minimizer $y^{*}$ for it.Step 7. If $\mathrm{rank}M_{t-k}\left(y^{*}\right)\neq \mathrm{rank}M_{t}\left(y^{*}\right)$, let k=k+1 and return to Step 6; if not, $\lambda_{i+1}=f_{i+1}^{k}$ and return to Step 3 with i=i+1.

A concrete example below is provided to illustrate Algorithm 1. Consider the symmetric tensor $A\in R^{\left[4,3\right]}$ such that

$$\begin{array}{ccc} A_{1111}=0.2147, & A_{1112}=-0.3147, & A_{1113}=-0.6738,\\ A_{1122}=0.1980, & A_{1123}=0.1335, & A_{1133}=-0.7441,\\ A_{1222}=0.0761, & A_{1223}=0.3524, & A_{1233}=-0.6900,\\ A_{1333}=-0.5758, & A_{2222}=-0.3686, & A_{2223}=0.3073,\\ A_{2233}=0.2145, & A_{2333}=0.0127, & A_{3333}=-0.7286. \end{array}$$

Using Algorithm 1, we can obtain all the Z-eigenvalues of A. The Z-eigenvalues are shown in Table 1, where $\lambda_{k}^{\left(n\right)}$ means $\lambda_{k}$ has n repeated roots and $n=\mathrm{rank}M_{t}\left(y^{*}\right)$. The computation task takes about 4.56 s.

#### 3.2.2. Homotopy Continuation-Type Algorithm

Compared with other calculation algorithms, the homotopy method has its own advantages, like less restrictive conditions, fewer operation costs, more universal methods to compute all common types of tensor eigenvalue, and so on. In order to use the homotopy method, we define the equivalence class

(15) $$T\left(\lambda ,x\right)=\left(\begin{array}{c} {\left(A^{\left(k\right)}x^{m-1}\right)}_{1}-\lambda {\left(Bx^{m^{\prime }-1}\right)}_{1}\\ \vdots \\ {\left(A^{\left(k\right)}x^{m-1}\right)}_{n}-\lambda {\left(Bx^{m^{\prime }-1}\right)}_{n}\\ a_{1}x_{1}+a_{2}x_{2}+\cdots +a_{n}x_{n}+b \end{array}\right)=0,$$

where $A\in R^{\left[m,n\right]}$, $B\in R^{\left[m^{\prime },n\right]}$, λ and $x={\left(x_{1},\cdots ,x_{n}\right)}^{T}$ are unknown, while $a_{1},\ldots ,a_{n},b$ are random numbers.

The main idea of the homotopy method is to convert the general polynomial system T(x)=0 into another polynomial system Q(x)=0 that is easy to solve. In particular situations, the homotopy H(x,t)=0 has smooth solution paths parameterized by *t* for t∈[0,1), and all the isolated solutions of P(x)=0 can be reached by tracing these solution paths. A useful homotopy is the linear homotopy [50]:

(16) H(x,t)=(1−t)γQ(x)+tT(x)=0,t∈[0,1],

where γ is a random nonzero complex number. Another common homotopy is the polyhedral homotopy [51,52] based on Bernstein’s theorem:

(17) $$H(x,t)=\left(h_{1}\left(x,t\right),\ldots ,h_{n}\left(x,t\right)\right)=0,\;t\in \left[0,1\right],$$

where $h_{i}\left(x,t\right)=\left(1-t\right)\gamma Q_{i}\left(x\right)+tT_{i}\left(x\right)$ and $T_{i}\left(x\right)$ is the i-th polynomial in a polynomial system.

We mainly focus on the homotopy method to solve the tensor E-eigenvalue computing problem. The algorithm for computing the E-eigenvalue by the homotopy method is provided as follows. Firstly, with the polyhedral homotopy method, we can obtain the equivalence class T(λ,x). The solution of T(λ,x) is relevant to the tensor eigenpairs, and (λ,x) is called equivalence eigenpairs. The polynomial expression T(λ,x) for the E-eigenvalue is

(18) $$T\left(\lambda ,x\right)=\left(\begin{array}{c} {\left(A^{\left(1\right)}x^{m-1}\right)}_{1}-\lambda x_{1}\\ \vdots \\ {\left(A^{\left(1\right)}x^{m-1}\right)}_{n}-\lambda x_{n}\\ a_{1}x_{1}+a_{2}x_{2}+\cdots +a_{n}x_{n}+b \end{array}\right)=0,\;$$

where $A\in R^{\left[m,n\right]}$, B is the identity matrix $I_{n}\in R^{n\times n}$. Then, we obtain all equivalent eigenvalues and eigenvectors from the equivalence class by using NAClab [53] based on the PSolve [54] method, which is widely applied in sparse matrix factorization such as [55,56]. NAClab, a Matlab toolbox realizing the PSolve method, provides us a package in the numerical solution of polynomial systems by the homotopy continuation method. Finally, we find all E-eigenpairs by using the correspondence between equivalence eigenpairs and E-eigenpairs.

The implementation of the algorithm is shown in Algorithm 2 [34].

Some concrete examples are given below. Consider the symmetric tensor $A\in R^{\left[4,3\right]}$, which is same as the example A given in the semidefinite relaxation algorithm. Using Algorithm 2, we likewise obtain all the E-eigenvalues and E-eigenvectors of A. Then, we can filter the real part of E-eigenpairs to obtain Z-eigenpairs. The Z-eigenvalues are shown in Table 2. The computation task takes about 0.28 s, which is a significant reduction in calculation time compared with the previous algorithm.
**Algorithm 2** Compute all E-eigenpairs of AStep 1. Using modified PSolve to obtain all equivalent eigenvalues and eigenvectors (λ,x) of T(λ,x).Step 2. For each (λ,x) obtained in Step 1, if $x^{T}x\neq 0$, normalize it to obtain an eigenpair $\left(\lambda^{*},x^{*}\right)$ by
$$\lambda^{*}=\frac{\lambda }{{\left(x^{T}x\right)}^{\left(m-2\right)/2}},\;x^{*}=\frac{x}{{\left(x^{T}x\right)}^{1/2}}$$.Step 3. Compute all E-eigenpairs $\left(\lambda^{\prime },x^{\prime }\right)$ of $\left(\lambda^{*},x^{*}\right)$ by $\lambda^{\prime }=t^{m-2}\lambda^{*}$ and $x^{\prime }=tx^{*}$ with t=±1.

Then, consider the general tensor $B\in R^{\left[3,4\right]}$ such that

$$B_{1}=\left[\begin{array}{cccc} 0.8810 & 1.8586 & 0.1136 & 1.4790\\ 0.3232 & -0.6045 & -0.9047 & -0.8608\\ -0.7841 & 0.1034 & -0.4677 & 0.7847\\ -1.8054 & 0.5632 & -0.1249 & 0.3086 \end{array}\right],$$

$$B_{2}=\left[\begin{array}{cccc} -0.2339 & -1.4694 & 0.3362 & -1.8359\\ -1.0570 & 0.1922 & -0.9047 & 1.0360\\ -0.2841 & -0.8223 & -0.2883 & 2.4245\\ -0.0867 & -0.0942 & 0.3501 & 0.9594 \end{array}\right],$$

$$B_{3}=\left[\begin{array}{cccc} -0.3158 & 0.9407 & -0.5583 & -0.9087\\ 0.4286 & 0.7873 & -0.3114 & -0.2099\\ -1.0360 & -0.8759 & -0.5700 & -1.6989\\ 1.8779 & 0.3199 & -1.0257 & 0.6076 \end{array}\right],$$

$$B_{4}=\left[\begin{array}{cccc} -0.1178 & -1.4831 & 1.1287 & 1.1741\\ 0.6992 & -1.0203 & -0.2900 & 0.1269\\ 0.2696 & -0.4470 & 1.2616 & -0.6568\\ 0.4943 & 0.1097 & 0.4754 & -1.4814 \end{array}\right],$$

where $B_{n}$ is the n-th slice of B along the third order. By using Algorithm 2, we obtain all the E-eigenvalues and E-eigenvectors of B. The E-eigenvalues are shown in Table 3. The computation task takes about 0.37 s.

#### 3.2.3. Calculation Performance Analysis

In this section, we will evaluate the calculation performances of the above two algorithms from several different aspects, such as the ratio of convergence, accuracy, and CPU time. All the numerical experiments were conducted on a PC with an Intel(R) core (TM) CPU at 3.00 GHz, 8 GB of RAM, and Windows 10. The packages Tensor-Toolbox [9] and TenEig-2.0 [34] were run using Matlab 2020a.

Firstly, the function “tenrand” in Tensor-Toolbox was used to generate the third-order two-dimension tensors to the fifth-order three-dimension tensors with 5000 sets across a total of 50,000 sets, in which the values of tensors follow the standard normal distribution. The generated dataset used Algorithm 2, “eeig” in TenEig-2.0, to calculate all the E-eigenpairs. As a matter of convenience, the non-convergence ratio, which is used to characterize the ratio of convergence, is defined as the number of error tensors, reporting the error during the computing over the total number of tensors. Among the 50,000 sets of data in this experiment, there were four tensors that did not converge. The non-convergence ratio is $8\times 10^{-5}$, and the specific occurrence of non-convergence is shown in the following Table 4.

The accuracy of the algorithm is divided into an estimation bias, which is known as residual, and upper bound bias. The residual is defined as the difference between the actual truth value and the estimated fit value, that is, the value of $Xx_{i}^{m}-\lambda_{i}$, where $X\in R^{\left[m,n\right]}$ is an arbitrary tensor and $\left(\lambda_{i},x_{i}\right)$ is the eigenpair of X. For E-eigenpairs of $X\in R^{\left[m,n\right]}$, the upper bound is $\left({\left(m-1\right)}^{n}-1\right)/\left(m-2\right)$ [57], which means that X has $\left({\left(m-1\right)}^{n}-1\right)/\left(m-2\right)$ equivalent eigenpairs if the number of E-eigenpairs are finite, and we use the notation E[m,n] to represent the upper bound. We use the PSolve method in Algorithm 2 to obtain equivalent eigenpairs directly, and in the third step of the algorithm, the equivalence eigenpairs are transformed into eigenpairs, so the actual upper bound in theory is 2E[m,n], which is called $E_{ac}\left[m,n\right]$. Based on the previous ratio of convergence test, the residual and upper limit data are obtained at the same time.

From Table 5, we can see that the residual is roughly 15 decimal places, which is negligible. The upper bound in theory is the same as the calculated upper bound. Finally, the computing costs represented by CPU time are shown in Table 6.

For small-scale tensors, where the order and dimension are less than four, the computing cost is linearly dependent on the upper bound with each eigenpair costing approximately 0.01 s. For large-scale tensors, affected by the “curse of dimension”, the upper bound increases rapidly, and the cost of computation increases extremely fast. The upper bound of the fifth-order five-dimension tensor is 682, and the CPU time is 30.28 s, while the upper bound of the sixth-order six-dimension tensor is 7812 and the CPU time is increased to about 2878 s, where the unit cost increases to 0.37 s.

## 4. Spectrum Situation Awareness Based on Tensor Eigenvalues

Spectrum situation awareness can be divided into three stages: perception (sensing), understanding, and prediction. Spectrum perception is the primary task to know the spectrum usage situation, which can be fulfilled by various sensors. Due to the unideal environment with noise, it is necessary to use heterogeneous information to improve sensing performance.

For SAGINs with the tensor big data model, it is indispensable to solve two significant problems before the spectrum situation awareness; one is the storage overhead problem, and the other is the data missing problem. These two problems can be well solved by tensor decomposition and tensor completion, which were mentioned above. For a big data tensor $A\in R^{n_{1}\times n_{2}\times \ldots \times n_{m}}$, the required storage space is $n^{m}$, and the overhead is unacceptable when *m* and *n* are large. Based on this, CP decomposition can greatly reduce the storage overhead. The specific algorithm [9] is as Algorithm 3.

In Algorithm 3, ★, ⊙, and ${\left(\cdot \right)}^{†}$ are the Hadamard product, Khatri–Rao product, and the Moore–Penrose pseudoinverse, respectively. $A_{j}$ is the j-mode unfolding of A. ||·|| is the tensor norm, taking A as an example, $\left||A|\right|=\sqrt{\sum_{i_{1},\ldots ,i_{m}=1}^{n_{1},\ldots ,n_{m}}{\left(A_{i_{1},\ldots ,i_{m}}\right)}^{2}}$. The space overhead for storing the big data tensor A is reduced to mnR.
**Algorithm 3** CP decomposition algorithm for big data tensorsInput: the big data tensor A, the CP-rank R, the tolerance $\gamma_{0}$, and the maximium number of iterations $N_{0}$.Step 0. Initialize $A_{i}\in R^{n_{i}\times R}$ for i=1,…,m, j=0, and N=1.Step 1. Compute $A_{j}=A^{\left(j\right)}\left(A_{m}⊙\cdots ⊙A_{j+1}⊙A_{j-1}⊙\cdots ⊙A_{1}\right)({A_{1}}^{T}A_{1}★\cdots ★{A_{j-1}}^{T}A_{j-1}★{A_{j+1}}^{T}A_{j+1}★\cdots ★{A_{m}}^{T}A_{m}{)}^{†}$, and j=j+1.Step 2. If j<=m, return to Step 1. Otherwise, compute $\gamma =1-\frac{||A-A_{1}\circ \cdots \circ A_{m}\left|||\right|}{\left||A|\right|}$.Step 3. If $\gamma <\gamma_{0}$ and $N<N_{0}$, let j=1, N=N+1, and return to Step 1.Output: $A_{1},\ldots ,A_{m}$.

In the actual scenario, it is inevitable to avoid partial data loss because of sensor failure, transmission loss, and other reasons, which is particularly common for big data. For spectral big data tensor X, the missing value problem can be solved by tensor completion based on CP decomposition, Tucker decomposition, and the minimum trace norm. Representation by the optimization problem can be written

(19) $$\min_{X,A_{1},\ldots ,A_{m}}||X-A_{1}\circ \cdots \circ A_{m}{\left|\right|}^{2}\;s.t.\;X_{\Theta }=Y_{\Theta },$$

(20) $$\min_{X,G,A_{1},\ldots ,A_{m}}||X-G\times_{1}A_{1}\cdots \times_{m}A_{m}{\left|\right|}^{2}\;s.t.\;X_{\Theta }=Y_{\Theta },$$

(21) $$\min_{X}{\left||X|\right|}_{*}\;s.t.\;X_{\Theta }=Y_{\Theta },$$

where Θ is the set of nonzero-valued indexes in X, and $\left|\right|\cdot {\left|\right|}_{*}$ is the tensor trace norm, which is defined as ${\left||X|\right|}_{*}=\sum_{i=1}^{m}\alpha_{i}\left|\right|X^{\left(i\right)}{\left|\right|}_{*}$ with $\sum_{i=1}^{m}\alpha_{i}=1$, which can be regarded as the weighted sum of the n-mode unfolding matrix traces. All of these problems can be solved using the block coordinate descent algorithm, using the solution of (19) as an example to illustrate the tensor completion algorithm, as shown below Algorithm 4.
**Algorithm 4** CP-based completion algorithm for big data tensorsInput: the big data tensor $Y\in R^{n_{1}\times \cdots \times n_{m}}$, the tolerance $\gamma_{0}$, and the maximium number of iterations $N_{0}$.Step 0. Initialize $A_{1}\cdots A_{m}$ by using random numbers, Θ and its complementary set $\hat{\Theta }$, let $X_{\Theta }=Y_{\Theta }$, N=0 and k=1, and compute $\gamma_{k}=||X-A_{1}\circ \cdots \circ A_{m}{\left|\right|}^{2}$.Step 1. Let $X_{\hat{\Theta }}={\left(A_{1}\circ \cdots \circ A_{m}\right)}_{\hat{\Theta }}$.Step 2. Compute $A_{1}\cdots A_{m}$ of X by using Algorithm 3, and k=k+1.Step 3. Compute $\gamma_{k}=||X-A_{1}\circ \cdots \circ A_{m}{\left|\right|}^{2}$.Step 4. If $\gamma_{0}>\gamma_{k}-\gamma_{k-1}$ and $N<N_{0}$, return to Step 1.Output: the completed tensor X.

After solving the above problems, we try to use tensor eigenvalues to construct a spectrum situation awareness scheme. To the best of our knowledge, it is the first to use tensor eigenvalues to evaluate spectrum situation awareness. Similar to classical signal detection methods, the proposed situation awareness scheme is based on a binary hypothesis test

(22) $$x\left(t\right)=\left\{\begin{array}{cc} s(t)+n(t), & H_{1}\\ n(t), & H_{0} \end{array}\right.$$

where x(t) denotes the received signal, s(t) is the target signal, and n(t) is the noise. Hypothesis test results are $H_{1}$ and $H_{0}$.

Based on the spectrum situation awareness model, the signal tensor is generated with the target signal and noise. The eigenvalues of the signal tensor are used to construct the detectors, and the sensing results are generated by comparing the detector with the given threshold. For a given specific false alarm, the thresholds can be determined by the signal tensor with only noise $H_{0}$. In polynomial time, the eigenvalue of the signal tensor is calculated with the homotopy method. Then, the detection performances can be evaluated by comparing such an eigenvalue with the given threshold. If the detector is greater than the threshold, the state is $H_{1}$, indicating that there is a target signal. Otherwise, the state is $H_{0}$, indicating no signal.

**Figure 3:**
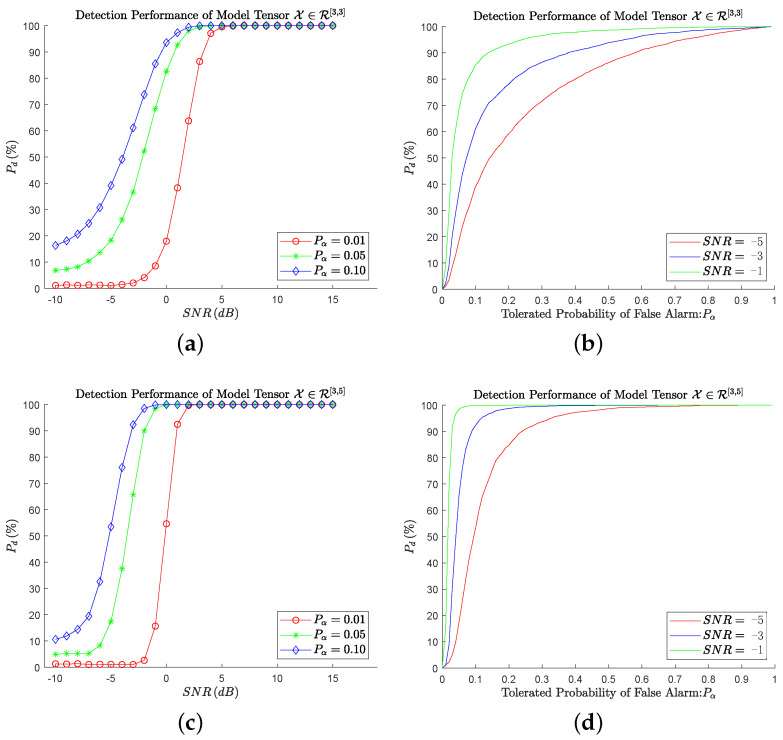
Probability of detection of spectrum sensing algorithms based on tensor eigenvalue, as a function of the SNR and the tolerated probability of false-alarm. (**a**) Performance against SNR; (**b**) performance against $P_{\alpha }$; (**c**) performance against SNR; (**d**) performance against $P_{\alpha }$.

In order to demonstrate the performance of the algorithm, we simulated 52 sets of spectrum tensors with different tensor sizes and SNRs by Matlab; each set consisted of 10,000 received signal tensors and 1000 noise tensors. The detection performances of Algorithm 5 are shown in Figure 3 with varying tensor sizes, SNRs, and $P_{\alpha }$. In Figure 3a, the probability of detection $P_{d}$ over 25 samples is plotted against the SNR under the various tolerated probabilities of false-alarm $P_{\alpha }$. It is found that with the increase of SNR, $P_{d}$ gradually increases and finally reaches 100%. For the same threshold, as the SNR increases, the maximum eigenvalue increases, and the detection probability also increases. This phenomenon shows that this algorithm is effective for spectrum detection. When the SNR is sufficient, the detection success probability is close to 100%; that is, the maximum eigenvalue can be used to represent the existence of average energy in the tensor. Moreover, we noticed at the same time that $P_{\alpha }$ mainly affects the speed of $P_{d}$ to reach 100%. The larger $P_{\alpha }$ is, the smaller SNR is when $P_{d}$ reaches 100%. When $P_{\alpha }$ = 0.01, SNR = 5 dB, that $P_{d}$ reaches 100% for the first time, but when $P_{\alpha }$ = 0.10, SNR = 2 dB. The reason for this phenomenon is that $P_{\alpha }$ directly affects the value of the threshold. When $P_{\alpha }$ increases, the threshold decreases, resulting in the tensor eigenvalue of a lower SNR being higher than the threshold.
**Algorithm 5** Algorithm of eigenvalue-based spectrum situation awarenessInput: the noise tensors N, the signal tensors X, and the tolerated probability of false alarm $P_{\alpha }$.Step 0. Compute the E-eigenvalues of N by Algorithm 2, and obtain the noise eigenvalue distribution.Step 1. Compute the threshold *T* for a given $P_{\alpha }$ based on noise eigenvalue distribution.Step 2. Compute the E-eigenvalues of X and obtain the maximum eigenvalue (module value) *S*.Step 3. Compare *T* and *S*. If T≥S, consider X to be the noise tensor. Otherwise, X is the signal tensor.Output: the result of spectrum situation awareness.

Based on the above findings, the detection probability will increase if the tolerated probability of false alarm increases and vice versa. In order to illustrate this effect, the algorithm is compared in terms of the probability of detection $P_{d}$ as a function of the tolerated probability of false alarm $P_{\alpha }$ in Figure 3b. When $P_{\alpha }<0.15$, $P_{d}$ increases rapidly, while when $P_{\alpha }>0.15$, $P_{d}$ increases gradually, and the curve of the larger SNR is above that of the smaller. The former is because, in hypothesis testing, the threshold distribution obeys the Gaussian distribution. In the first part, the threshold decreases rapidly with the increase of $P_{\alpha }$, and the detection success probability increases rapidly with the same SNR. In the second part, due to the concentrated distribution of the threshold, the threshold changes insignificantly with the increase of $P_{\alpha }$, resulting in a slow change of detection probability. The latter is because the larger SNR makes the desired threshold for $P_{d}=100\%$ higher, which is self-consistent with the conclusion drawn by Figure 3a.

Figure 3c,d are mainly to illustrate the effect of tensor size on the algorithm. Instead of using tensors $X\in R^{\left[3,3\right]}$ in Figure 3a,b, we use tensors $Y\in R^{\left[3,5\right]}$ in Figure 3c,d, where each tensor grows from 27 elements to 243 elements. From the comparison, it is easy to find that the effect of the algorithm for Y is significantly better than that for X. When $P_{\alpha }$ = 0.01, the SNR just has to be equal to 2 dB in order for $P_{d}$ to reach 100%. The slope of the front part of the curve is significantly higher than that of Figure 3a.

Through subsequent simulation experiments on tensors of different order and different dimensions, it seems to be concluded that for tensors of the same order, the larger the dimension is, the better the algorithm effect will be. For the same dimensional tensor, the larger the order is, the worse the algorithm is. Explaining the cause of this phenomenon is the key problem to be solved in the following work. However, in a word, the eigenvalue detection method for spectrum sensing can effectively solve the problem of spectrum signals.

## 5. Conclusions

A novel tensor-based spectrum situational awareness scheme has been proposed, and the tensor was used to model and process multidimensional, heterogeneous, and massive spectral big data from space–air–ground integrated networks. In particular, the tensor eigenvalues have been utilized to construct the spectrum situation awareness, in which the distributions of E-eigenvalues were included to formulate spectrum sensing algorithms. Two tensor eigenvalue calculation schemes have also been provided to generate tensor eigenvalues. Simulation results have evaluated the correctness of the proposed situational awareness scheme. The situation awareness scheme can detect the spectrum state quickly, accurately and coarse grained, providing valuable support for subsequent understanding and prediction. However, as the complexity of tensor eigenvalue computation increases catastrophically with the increase of tensor size, this scheme can only deal with local small-scale data of spectral big data. In the future, the research will focus on exploring the correlation between tensor decomposition and eigenvalue, aiming to enable large-scale tensor eigenvalue computation. Additionally, further investigation in tensor computing will be conducted to enhance the subsequent operations of situational awareness. Overall, the novel tensor-based spectrum situational awareness scheme has provided a new application paradigm for tensor theory.

## Figures and Tables

**Table 1:** Z-eigenvalues of $A\in R^{\left[4,3\right]}$ by semidefinite relaxation algorithm.

*k*	1	2	3	4
$\lambda_{k}$	$1.1878^{\left(2\right)}$	$0.5609^{\left(2\right)}$	$0.4543^{\left(2\right)}$	$0.3269^{\left(4\right)}$
*k*	5	6	7	8
$\lambda_{k}$	$-0.0584^{\left(2\right)}$	$-0.6287^{\left(2\right)}$	$-1.6377^{\left(2\right)}$	$-2.7482^{\left(2\right)}$

**Table 2:** Z-eigenvalues of $A\in R^{\left[4,3\right]}$ by homotopy continuation.

*k*	1	2	3	4
$\lambda_{k}$	$1.1878^{\left(2\right)}$	$0.5609^{\left(2\right)}$	$0.4543^{\left(2\right)}$	$0.3269^{\left(4\right)}$
*k*	5	6	7	8
$\lambda_{k}$	$-0.0584^{\left(2\right)}$	$-0.6287^{\left(2\right)}$	$-1.6377^{\left(2\right)}$	$-2.7482^{\left(2\right)}$

**Table 3:** E-eigenvalues of $B\in R^{\left[3,4\right]}$ by semidefinite relaxation algorithm.

*k*	1	2	3	4
$\lambda_{k}$	±(1.5909+0.0000i)	±(1.3095+0.0000i)	±(1.2097+0.0000i)	±(1.1606+1.4460i)
*k*	5	6	7	8
$\lambda_{k}$	±(1.1606−1.4460i)	±(1.0632+1.0201i)	±(1.0632−1.0201i)	±(0.8579+1.1735i)
*k*	9	10	11	12
$\lambda_{k}$	±(0.8579−1.1735i)	±(0.8360+0.0260i)	±(0.8360−0.0260i)	±(0.6803+1.6365i)
*k*	13	14	15	
$\lambda_{k}$	±(0.6803−1.6365i)	±(0.6331+0.0000i)	±(0.3802+0.0000i)	

**Table 4:** The non-convergence number in 5000 sets of tensors by homotopy continuation algorithm.

[m,n]	[3,2]	[3,3]	[3,4]	[3,5]	[4,2]
Num	0	0	2	0	0
[m,n]	[4,3]	[4,4]	[4,5]	[5,2]	[5,3]
Num	1	0	0	1	0

**Table 5:** The accuracy in 5000 sets of tensors by the homotopy continuation algorithm.

(m,n)	(3,2)	(3,3)	(3,4)	(3,5)	(4,2)
residual	$5.30\times 10^{-16}$	$3.54\times 10^{-16}$	$3.12\times 10^{-16}$	$1.03\times 10^{-15}$	$3.30\times 10^{-16}$
$E_{ac}$	6	14	30	62	8
*N*	6	14	30	62	8
(m,n)	(4,3)	(4,4)	(4,5)	(5,2)	(5,3)
residual	$1.03\times 10^{-15}$	$1.90\times 10^{-15}$	$8.81\times 10^{-15}$	$5.85\times 10^{-16}$	$1.64\times 10^{-15}$
$E_{ac}$	26	80	242	10	42
*N*	26	80	242	10	42

**Table 6:** The time of computing in 5000 sets of tensors by homotopy continuation algorithm.

(m,n)	(3,2)	(3,3)	(3,4)	(3,5)	(4,2)
Time(s)	$8.79\times 10^{-2}$	$1.38\times 10^{-1}$	$2.59\times 10^{-1}$	$6.40\times 10^{-1}$	$9.86\times 10^{-2}$
(m,n)	(4,3)	(4,4)	(4,5)	(5,2)	(5,3)
Time(s)	$2.46\times 10^{-1}$	$8.16\times 10^{-1}$	4.58	$1.18\times 10^{-1}$	$4.11\times 10^{-1}$

## Data Availability

Data are contained within the article.
